# A Comprehensive Toxicological Assessment of Fulvic Acid

**DOI:** 10.1155/2020/8899244

**Published:** 2020-12-16

**Authors:** Chongshan Dai, Xilong Xiao, Yonglei Yuan, Gaurav Sharma, Shusheng Tang

**Affiliations:** ^1^College of Veterinary Medicine, China Agricultural University, No. 2 Yuanmingyuan West Road, Beijing 100193, China; ^2^Advanced Imaging Research Center, University of Texas Southwestern Medical Center, Dallas, TX 75390, USA

## Abstract

Fulvic acid (FA), a humic substance, has several nutraceutical properties, including anti-inflammation, antimicrobial, and immune regulation abilities. However, systematic safety assessment remains insufficient. In the present study, a battery of toxicological studies was conducted per internationally accepted standards to investigate the genotoxicity and repeated-dose oral toxicity of FA. Sprague-Dawley (SD) rats or ICR mice were used. Compared to the control group, there were no significant changes (all *p* > 0.05) in all FA treatment groups in the bacterial reverse mutation test, *in vitro* mammalian chromosome aberration test, *in vivo* sperm shape abnormality assay, and *in vivo* mouse micronucleus assay. The acute toxicity test showed that no mortality or toxic effect was observed following oral administration of the maximum dose of 5,000 mg/kg BW/day to mice or rats. A 60-day subchronic study was conducted at 0 (control), 200, 1,000, and 5,000 mg/kg/day. Compared to the control group, there were no significant changes (all *p* > 0.05) in the body weights, feed consumption, clinical signs, hematology, clinical chemistry, organ weights, or histopathology examinations. In conclusion, the no-observed-adverse-effect-level (NOAEL) of FA supplementation from the 60-day study was determined to be 5,000 mg/kg body weight/day, the highest dose tested. Our findings suggest that the oral administration of FA may have higher safety.

## 1. Introduction

The use of antibiotics in animal production has led to a dramatic decline in the prevalence of diseases; however, issues with medication residues have hindered the implementation of antibiotic treatment regimens. Currently, health management and biosecurity represent important practices in animal production [1, 2]. Humic acid (HA) is a natural organic product derived by chemical and biological processes from dead plants and animal tissues. HA is produced from the organic materials of dead plants and animal tissues by chemical and biological processes [3]. HA possesses various biology functions, including antioxidant, anti-inflammatory, immune-stimulatory, and antimicrobial properties, which have been used in agriculture, even as a supplementation used in human and animal clinical practices [4–9]. Studies indicated that the HA has been used as the neuron- or nephron-protective agents in clinical therapy based on the potential anti-inflammatory ability [10, 11]. An earlier study demonstrated that HA exhibited the potent activity against human immunodeficiency virus that is not treated due to lack of the effective drugs [12], whereas, in veterinary medicine, HA may cure certain clinical symptoms including diarrhea, dyspepsia, and acute intoxication in horses, ruminants, swine, and poultry [3, 6]. HA has also recently been used to increase the growth rate and improve feed efficiency and immunity, as well as improve the economic and ecological benefits during animal production [9, 13].

Fulvic acid (FA), one fraction of HA, has a lower molecular weight and a higher oxygen content than other HA components [3]. Several studies showed that FA is a potential safe natural active ingredient of HA [14, 15]. As a dietary supplementation, FA can be found in a liquid form as a component of mineral colloids. It has been reported that FA has many reactive functional groups, including carboxyls, hydroxyls, phenols, quinones, carbonyls, and semiquinones, which possess the potent activities in metal removal and anti-inflammatory capabilities [3]. A recent study has shown that FA can protect against homocysteine-induced inflammation by targeting inhibition of cyclooxygenase-2 expression in human monocytes [16]. Some studies also demonstrated that FA could inhibit the aggregation of Tau fibrils associated with Alzheimer's disease [17, 18]. In animal production, many reports indicated that the FA could be used as a feed additive, protect against infections and poisoning, and improve utilization of nutrients, growth performance, and meat quality of producing animals [19–21]. Besides, FA could also be used to treat the infections caused by drug-resistant pathogens [14, 15, 22]. Although the previous studies had shown that HA may have the potential genotoxicity through *in vitro* testing [23, 24], there is very little information on the toxicological effect of FA. A previous study showed that FA treatment at 1.58–2.43 *μ*g/*μ*L for 48 h or 72 h could inhibit cell proliferation and induce the gene expression of the apoptosis pathway in Hep3B, HT29, and PC3 cells [25]. Also, it has been argued that FA treatment at 5 *μ*g/mL for 30 min can induce inflammatory response by activating nuclear factor-*κ*B (NF-*κ*B) pathway in RAW 264.7 cells [26]. Therefore, with the increasing application of FA, its safety assessment remains required. In this study, the acute toxicity and subchronic toxicity tests (60-day repeat-dose oral toxicity studies) were conducted in mice or rats. Furthermore, various genotoxicity tests, including *in vitro* bacterial reverse mutation assay, *in vivo* mouse bone marrow micronucleus assay, and sperm shape abnormality assay, were conducted according to international and FDA standards. Our current study will provide more information for the toxicological effects of FA, which will be useful for its application.

## 2. Materials and Methods

### 2.1. Materials

The FA used in the current experiment was extracted from weathered coal obtained. The effective content of FA was determined by Inner Mongolia Yongye Nongfeng Biotechnology Co. Ltd. (Inner Mongolia, China) according to the published literature [19], and the purity was 12.05%. Carboxyl methylcellulose sodium (CMC-Na) and cyclophosphamide (CP) were purchased from Tianjin Chemical Reagent Company (Tianjin, China). Phenobarbital/benzoﬂavone-induced (10%) rat liver S_9_ was purchased from Platt Bio-Pharmaceutical Co., Ltd. (Wuhan, China). 2-Aminoﬂuoren, sodium azide, and p-dimethylaminobenzenediazosodium sulfonate were purchased from sigma (Louis, MO, USA). FA suspensions were prepared with 0.5% of CMC-Na based on the concentration administered prior to oral gavage.

### 2.2. Animals

All procedures performed on the experimental animals were approved by the China Agricultural University Animal Care and Use Committee. Female- and male-specific pathogen-free (SPF) Sprague-Dawley (SD) rats and ICR mice were purchased from Vital River Animal Technology Co., Ltd. (Beijing, China) (laboratory animal reproduction license #SCXK (B) 2012-0001). All animals were housed individually at 22 ± 3°C, a relative humidity of 50 ± 10%, and a 12 h light/dark cycle. Rats and mice were fed ad libitum during the one-week acclimatization period.

**Figure 1:**
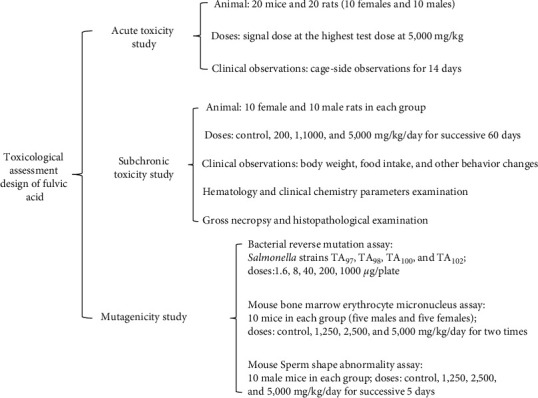
Experimental design of toxicological assessment of fulvic acid.

In the current study, all experimental designs strictly followed guidelines of Organization for Economic Cooperation and Development (OECD), FDA or Chinese standard [27–29], and a protocol showed in Figure 1.

### 2.3. Acute Toxicity Study

SD rats (8 weeks old, 180–220 g) and ICR mice (8 weeks old, 22-25 g) were used in acute toxicity tests and the protocol was carried out in accordance with guidelines No. 423 and 425 of OECD [30]. 10 male and 10 female ICR mice or SD rats were administrated with 5,000 mg/kg FA via oral gavage. The mice or rats in the control group were treated with an equal volume of 0.5% CMC-Na. After signal administration, surviving animals were observed for 14 days. Cage-side observations were performed, that is, evaluation of skin and fur, respiratory, eyes, and mucous membranes, autonomic effects (e.g. salivation), gait and posture, central nervous system effects (e.g., tremors and convulsions), and behavioral changes (e.g., level of motor activity and reactivity to handling and stereotyping or bizarre behavior). Besides, the time was also recorded as precisely as possible if the animal died. Finally, all the surviving animals were euthanized intraperitoneally with overdose of sodium pentobarbital at 80 mg/kg and quickly sacrificed for anatomical observation and all protocols were conducted under FDA's Good Laboratory Practice guidelines [27].

### 2.4. Subchronic Toxicity Study

#### 2.4.1. Dose Designs

According to the tolerance test of FA above, a 60-day subchronic toxicity test was designed in rats. Male and female rats (80–100 g) were randomly divided into four groups (0, 200, 1,000, or 5,000 mg/kg/day) by a computer-generated (weight-ordered) randomization procedure. Each group has consisted of 10 female and 10 male rats. The rats in the control group were given an equal volume of 0.5% CMC-Na equally as the vehicle control. All rats were treated with consequent 60 days and clinical observations were performed throughout the experiments. After an overnight fast (approximately 16 h) following final administration of the test article at the 30th or 60th day, five rats were anesthetized with pentobarbital sodium and killed and sacrificed, respectively. The blood and organs have been rapidly collected for the hematology, biochemical, and histopathological studies as described below.

#### 2.4.2. Clinical Observations

Bodyweight, behavior, and appearance were carefully observed and recorded during the experiment. All rats were observed at least once a day for mortality or morbidity and changes in posture, changes in the skin, fur, eyes, mucous membranes, and behaviors. In addition, a functional observation battery (FOB) was used during the final week to evaluate parameters such as general physical condition and behavior, response to handling, sensory reactions to various stimuli, grip strength, and motor activity. Measurements of body weight were recorded every five days and the body weight gain was analyzed. Food intake was determined and food efficiency calculated every five days.

#### 2.4.3. Hematology and Clinical Chemistry Parameters Examination

For the hematology examination, one part of blood samples was corrected and treated with ethylene diamine tetra-acetic acid (EDTA) to analyze the hematological parameters including red blood cell count (RBC), hemoglobin concentration (HG), blood platelet hematocrit (PLT), and white blood cell count (WBC) by a Coulter HmX Hematology Analyzer (Beckman Coulter Inc., Fullerton, CA, USA).

For the clinical chemistry parameter examination, another part of the blood sample was centrifuged at 3,000 g for 10 min and the supernatant was collected to examine clinical chemistry parameter by using a Synchron Clinical System CX4 (Beckman Coulter, Brea, CA, USA) according to the manufacturer's instructions (Beijing Leadman Biochemistry Technology Co. Ltd., Beijing, China). These parameters included alanine aspartate aminotransferase (AST), aminotransferase (ALT), protein (TP), albumin (Alb), total cholesterol (TCH), creatinine (Cr), and blood urea nitrogen (BUN).

#### 2.4.4. Gross Necropsy and Histopathological Examinations

The changes in all the organs/tissues were carefully examined macroscopically and the gross lesions were recorded. The organs including the liver, kidney, spleen, heart, lung, brain, ovary, uterus, prostate, adrenal glands, and testicles (epididymis) were rapidly separated and selected organ weights (absolute and relative) were conducted on all animals. Subsequently, all the organs except testes of each animal were fixed in 10% neutral buffered formalin for the light microscopy histological examination. The formalin-fixed tissue was embedded in paraffin, sectioned at 4 *μ*m, and then stained with hematoxylin-eosin (H&E) for the morphological alterations by a board-certified veterinary pathologist. Histopathological examinations were conducted for preserved organs and tissues of all control animals and at the highest dose (i.e., 5,000 mg/kg BW group) and for organs with gross lesions or other abnormalities in other groups.

### 2.5. Mutagenicity Studies

#### 2.5.1. Bacterial Reverse Mutation Assay

A bacterial reverse mutation assay was performed to evaluate the mutagenicity of FA, with and without S_9_, using the following four *Salmonella* strains TA_97_, TA_98_, TA_100_, and TA_102_, as prescribed in the OECD guideline No. 471 [28]. All strains were provided by the National Center for veterinary safety evaluation (Beijing, China). Five concentrations (e.g., 1000, 200, 40, 8, and 1.6 *μ*g/plate) were selected for the initial and confirmatory tests based on the preliminary test results. FA were prepared with 0.5% CMC-Na according to the required concentration in a constant volume. The control group was treated with the equal volume of 0.5% CMC-Na. 2-Aminoﬂuoren was used as a positive control for all strains tested with S_9_. Sodium azide was used as a positive control for TA_100_ strains tested without S_9_. p-dimethylaminobenzenediazosodium sulfonate was used as a positive control for testing TA_97_, TA_98_, and TA_102_ strains without the treatment of S_9_. The protocols were performed according to a Chinese standard [29]. Each experimental condition was performed in triplicate.

#### 2.5.2. Mouse Bone Marrow Erythrocyte Micronucleus Assay

Mice bone marrow erythrocyte micronucleus assay was performed per the OECD Guideline No. 474 [28] for principles of Good Laboratory Practices. Fifty SPF ICR mice (8 weeks old, 22–25 g) were randomly divided into five groups and 10 mice in each group (five males and five females). CP was intraperitoneal (i.p.) administered at a signal dose of 40 mg/kg BW at 6 h before sampling as a positive control [29] and 0.5% of CMC-Na was used as a negative control. In this dose-response study, FA was administered twice in 30 h with a 24 h interval at the dose of 1,250, 2,500, and 5,000 mg/kg BW through oral gavage. Six hours after the last treatment, all the animals were euthanized to obtain cell suspensions from the femur bone marrow. Also, all animals were observed immediately after dosing and at regular intervals until sacrifice (by cervical dislocation) for mortality, visible signs of toxicity, or other reactions to treatment. The frequencies of the micronucleus and the occurrence rate of the micronucleus were examined and recorded after a series of dying process and treatment. The ratio of polychromatic erythrocytes (PCE) to normochromatic erythrocytes (NCE) was determined for each animal by counting a total of 1000 erythrocytes.

#### 2.5.3. Mouse Sperm Shape Abnormality Assay

Sperm shape abnormality assay was performed following the principles of Good Laboratory Practices [27] and Chinese standard (GB15193.7-2003) [29]. Fifty SPF ICR mice (8 weeks old, 22–25 g) were randomly divided into five groups and 10 male mice in each group. Mice were orally administrated with FA at the doses of 1250, 2500, and 5000 mg/kg BW for the five days. The mice in the positive control were treated with CP (i.p. 40 mg/kg BW) for continuous five days [29]. 0.5 % CMC-Na was used as a negative control. On the 35th day, mice were sacrificed by cervical dislocation.

Smears were made for sperm morphology assay after a series of processing according to standard method (GB15193.7-2003) [29]. A total of 1000 sperms per animal were scored under a microscope with 40x magnification. Sperm head abnormalities were determined as having either normal or abnormal morphology. A “hookless head” does not have a spherical spot at the tip of the sperm head, a “banana head” has a banana-like shape, an “amorphous head” lacks the usual hook and is deformed, and a “folded sperm” is folded on itself.

### 2.6. Statistical Analyses

Unless specified, all results shall be shown as mean ± standard deviation (SD). A one-way analysis of variance, followed by a Fisher's LSD test, was used to compare any two means when the variance was homogeneous; otherwise, Dunnett's T3 test was used (SPSS Inc., Chicago, IL, USA). A *p* < 0.05 has been considered as statistical significance.

## 3. Results

### 3.1. Acute Toxicity

During the experiments, no animals died and no unexpected toxic signs or symptoms were found. Also, no changes were noted in the daily intake of feed and water and no significant changes in necropsy were observed during the corresponding 14-day monitoring after mice or rats received FA at the 5,000 mg/kg BW level; the estimated median lethal dose (LD_50_) of FA was over 5,000 mg/kg BW in rats and mice.

### 3.2. 60-Day Subchronic Toxicity Assay

Based on FA-LD_50_ in rats, the 60-day subchronic evaluation of toxicity was conducted and the findings are shown as follows.

#### 3.2.1. Clinical Observations

During the daily detailed clinical observations, there was no death and anomaly recorded in any animal's clinical signs, behavior, or physical condition. No statistically significant variations in overall and normal average body weight gain, food, and water consumption were found at various time intervals (Table 1 and Figure S1) and even the food efficiency (Table 2) was found consistent in all the dosage groups tested relative to control groups during the study.

#### 3.2.2. Hematology and Serum Biochemical Changes

As shown in Tables 3 and 4, no substantial differences (*p* > 0.05) were found in the overall dosage group of FA in hematology and serum biochemical parameters except for a small rise in PLT, BUN, and Cr in the 5,000 mg/kg BW/day group compared with the control group on the 30th and 60th day.

#### 3.2.3. Organ Index, Macroscopic Observation, and Histopathological Changes

On the 30th and 60th day, there were no significant changes in the organ index in all dose groups, compared to the control (data were not shown), whereas, the macroscopic examination of the organs or tissues including the liver, kidneys, spleen, heart, lungs, brain, ovary, uterus, prostate, adrenal glands, and testicles revealed no abnormal changes, relative to control group. In addition, histopathological modifications were examined at the highest dose level, that is, 5,000 mg/kg/day group, in major organs including liver, kidney, heart, spleen, lung, and intestinal tissue. No physiological variations were observed in comparison to the control group (Figure 1).

### 3.3. Mutagenicity

#### 3.3.1. Bacterial Reverse Mutation Assay

Analysis of *Salmonella* (*S.*) *typhimurium* reverse mutation in the presence or absence of a metabolic activation mechanism (S_9_) was performed and the findings were shown in Table 5. Compared to the corresponding negative control, no increase >2-fold in the number of reverse was observed with the *S. typhimurium* strains TA_97_, TA_98_, TA_100_, and TA_102_ after treatment with FA in the range of 1.6 *μ*g–1000 *μ*g/plate, but there was a substantial increase (>2-fold) in the positive control (2-aminofluorene, p-dimethylaminobenzidenediazo sulfonate, and sodium azide, respectively).

#### 3.3.2. *In Vivo* Mouse Bone Marrow Erythrocyte Micronucleus Assay

In this study, there was no death, evidence of toxicity, or gender-specific effects. As shown in Table 6, PCE/RBC radios and PCE micronucleus frequency had no statistically relevant changes in the doses of BW groups of 1,250, 2,500, and 5,000 mg/kg compared to the control group. In the positive control group, a significant statistical shift (*p* < 0.01) was observed in the frequency of the PCE micronucleus relative to the negative control **(**Table 6).

#### 3.3.3. Sperm Shape Abnormality Assay

As shown in Table 7, the rate of sperm abnormal morphology and proportion of malformation type was examined after FA treatments. Compared with the negative control group (0.5% CMC-Na), the sperm abnormality in all the FA treatment groups (i.e., 1,250, 2,500, and 5,000 mg/kg BW/day) showed no significant changes. In the positive control group (CP; 40 mg/kg), the radios of sperm abnormality significantly increased to 9.47 ± 1.96% (*p* < 0.01), compared to the control group.

## 4. Discussion

Over the last two decades, owing to a rise in antibiotic resistance and a scarcity of effective antibiotic medications, substitute antibiotic treatment has been proposed to be used in humans or as a growth promoter and has become a potential option. FA is the key active ingredient in HA, and it has been used in human medicine or as the animal feed additives in animal production on the basis of its various pharmacological properties and relatively safer compared to the direct use of antibiotics, such as colistin sulfate [31]. Our results revealed that the no-observed-adverse-effect-level (NOAEL) of FA from the 60-day study was determined to be the highest dose tested (5,000 mg/kg BW/day) in the present study.

**Figure 2:**
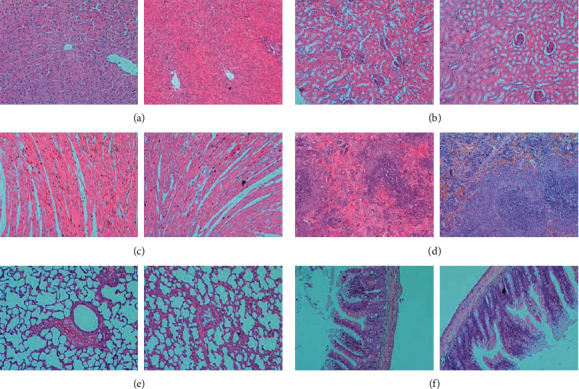
Representative histopathological changes of liver, kidney, heart, spleen, lung, and intestine tissues. The histopathological changes of major organs including liver (a), kidney (b), heart (c), spleen (d), lung (e), and intestine (f) tissues were examined in the control groups (left) and the highest dose group (right), that is, 5000 mg/kg/day group. Magnification 10×.

In an earlier study, no adverse effect and teratogenicity were observed in rats orally administrated with FA at the dose of 100 mg/kg BW/day for 183 days [32]. Interestingly, oral administration of FA ≥100 mg/kg effectively reduced carrageenan-induced paw edema in rats [32]. In another study, carbohydrate-derived FA showed potent activity against fungal pathogens with lower minimum inhibitory concentration (MIC) values (equal or less than 5 mg/mL), while the cytokine interleukin 6 mediated-inflammation response was significantly inhibited [14]. In the human clinical toxicity assessment study, FA is indicated to be safe in humans at a daily dosage of 1.8 g (equal to purified FA at 30 mg/BW/day) [5]. Addington et al. reported that it had no toxic signs, pathology changes of a gross organ, or animal death when the male and female adult rats were administered up to 4786 mg/kg of HA [33]. In our current study, no adverse effects were detected in mice or rats following an FA oral gavage at 5000 mg/kg BW/day (equal to a purified FA at 625 mg/BW/day) (Tables 1–7 and Figure 2).

There was a slight increase in body weight gain in all FA-treated groups between days 0 and 20 in female rats but a slight decrease in male rats compared to their respective controls (Figure S1). The gain of body weight and water had no significant changes intake in all FA-treated groups. Similarly, a recent study showed that the body weights, hematological variables, indices of thyroid function, and microscopic organ histology had no significant changes when adult rats were orally administrated with HA at the dose of 2000 mg/kg for 90 days [34]. In another study, dietary supplementation of 0.2%, 0.4%, and 0.6% FA significantly reduced mean backfat thickness of pigs with the significant increases of the serum levels of low-density lipoprotein, leptin, growth hormone, insulin, and triiodothyronine [20]. These data indicated that FA had higher safety, far beyond effective dose.

In a preclinical toxicity study, there was no adverse effect on the pups when pregnant female rats were orally administered with HA at 500 mg/kg BW/day for 1 month [35]. HA is suggested as a potential safe natural active substance [14, 22] and largely nontoxic and nonteratogenic [6, 13]. Some studies however suggested that Kashin-Beck disease in children and adolescents in East Asia is positively associated with the HA levels [36]. HA, at the dose of 50–100 *μ*g/mL, can induce chromosomal abnormalities in intestinal cells and marginal but nonsignificant induction of aneuploidy in bone marrow cells *in vitro* [37]. In another study, HA at the dose of 200 *μ*g/mL did not cause mutation in *Salmonella typhimurium* TA_97_, TA_98_, TA_100_, TA_102_, and TA_1535_ evaluated by the *Ames* test, but comet assay showed positive results [38]. These indicated that the different mutagenicity tests may present the different phenotype of genotoxicity. It was therefore concluded that the mutagenic thresholds of HA could be worked out by further research using different techniques, different biological materials, and different genetic endpoints. In the current study, we selected the *in vitro* bacterial reverse mutation assay, *in vivo* mammalian erythrocyte micronucleus assay, and mouse sperm abnormality test to together evaluate the genotoxicity of FA, as per FDA and OECD guidelines [27, 28]. Our findings from *in vitro* bacterial reverse mutation assay (Table 5), the mice bone marrow erythrocyte micronucleus assay (Table 6), and the sperm abnormality assay (Table 7) showed that FA had no genotoxic effect. Our results are consistent with the Addington studies [33]. FA has a lower molecular weight and more carboxyl in chemical structure than HA, which may contribute to explaining the differences between them in biological activities or toxic effects [22].

No toxicity reaction was observed in all the FA treatment groups under the specified experimental conditions. No evidence of mutagenicity was observed in a bacterial reverse mutation test or *in vivo* assay of the sperm abnormality shape, whereas no genotoxicity was observed in mouse *in vivo* micronucleus assays. The NOAEL of FA from the 60-day study was determined to be the highest tested dose (5, 000 mg/kg BW/day). Our findings indicated that the oral administration of FA may have higher safety.

## Figures and Tables

**Table 1:** The changes in total average daily food intake, water intake, and body weight gain during the 60-day repeated-dose oral toxicity studies of FA.

Index	Female				Male			
	Control	200 mg/kg BW	1, 000 mg/kg BW	5, 000 mg/kg BW	Control	200 mg/kg BW	1,000 mg/kg BW	5, 000 mg/kg BW
During 0-30 th day								
Total average food intake (g/day/rat)	12.04 ± 0.08	12.34 ± 0.09	12.32 ± 0.10	12.28 ± 0.08	15.58 ± 0.07	14.89 ± 0.08	15.26 ± 0.07	15.09 ± 0.14
Total average water intake (g/day/rat)	27.51 ± 0.70	28.19 ± 0.82	28.15 ± 0.88	28.06 ± 073	35.80 ± 0.61	34.21 ± 0.77	35.08 ± 0.67	34.69 ± 1.24
Total average daily body weight gain (g/day/rat)	3.52 ± 1. 19	3.65 ± 0.73	3.63 ± 0.73	3.55 ± 0.71	5.96 ± 1.00	5.70 ± 1.14	5.77 ± 1.15	5.74 ± 1.12

During 31st–60th day								
Total average food intake (g/day/rat)	22.74 ± 0.11	23.46 ± 0.29	23.17 ± 0.12	23.57 ± 0.16	35.00 ± 0.07	34.34 ± 0.29	34.00 ± 0.11	35.40 ± 1.17
Total average water intake (g/day/rat)	47.18 ± 0.46	48.67 ± 1.20	48.08 ± 0.51	48.90 ± 0.65	74.97 ± 0.30	73.53 ± 1.27	72.83 ± 0.45	75.79 ± 5.01
Total Average daily body weight gain (g/day/rat)	1.67 ± 0.83	1.72 ± 0.34	1.79 ± 0.36	1.95 ± 0.39	4.24 ± 0.56	4.33 ± 0.87	4.11 ± 0.82	4.09 ± 0.82

No statistically significant differences were found, compared to the corresponding control group.

**Table 2:** The effect on food efficiency in the 60-day repeated-dose oral toxicity studies of FA.

	Groups (mg/kg BW) (*n* = 10)	Body weight gain (g)	Feed uptake (g)	Feed efficacy rate (%)
Female	0.5% CMC-Na	106.2 ± 6.22	470.3 ± 12.19	22.58
	2 00	109.2 ± 5.07	494.3 ± 12.78	22.09
	1,000	114.4 ± 7.47	494.3 ± 12.79	23.14
	5,000	106.6 ± 4.36	489.5 ± 12.65	21.78

Male	0.5% CMC-Na	179.2 ± 7.39	604.2 ± 15.74	29.66
	200	169.6 ± 5.90	564.3 ± 14.68	30.05
	1,000	175.6 ± 5.32	599.2 ± 15.6	29.31
	5,000	167.8 ± 3.49	568.5 ± 14.79	29.52

No statistically significant differences were found. CMC-Na, carboxyl methylcellulose sodium.

**Table 3:** The effects of FA on hematology parameters of rats in the 60-day subchronic toxicity assay.

Parameters	HG (g/L)		RBC (M/mm^3^)		WBC (th/mm^3^)		PLT (th/mm^3^)	
	F	M	F	M	F	M	F	M
30-day								
0.5% CMC-Na	160.20 ± 6.97	158.60 ± 5.08	7.64 ± 0.53	7.66 ± 0.50	8.40 ± 0.59	8.15 ± 0.88	471.60 ± 67.92	465.00 ± 61.30
2 00 mg/kg BW	158.8 ± 4.83	153.2 ± 5.81	7.71 ± 0.35	7.38 ± 0.38	7.62 ± 0.5	8.61 ± 0.43	443.2 ± 71.3	461 ± 57.69
1 000 mg/kg BW	157.40 ± 7.94	157.00 ± 10.49	7.14 ± 0.42	7.03 ± 0.43	8.03 ± 0.83	8.20 ± 0.83	459.20 ± 58.31	449.80 ± 42.56
5 000 mg/kg BW	157.80 ± 6.85	152.80 ± 5.49	7.20 ± 0.59	7.77 ± 0.40	8.22 ± 0.81	7.98 ± 0.78	473.00 ± 66.21	453.80 ± 89.35

60-day								
0.5% CMC-Na	146.00 ± 9.86	151.60 ± 11.15	7.72 ± 0.53	8.01 ± 0.51	7.72 ± 0.53	8.09 ± 0.55	368.80 ± 67.42	368.60 ± 58.6
200 mg/kg BW	147.6 ± 9.2	153 ± 10.18	7.51 ± 0.5	7.76 ± 0.37	7.62 ± 0.5	7.76 ± 0.37	393.8 ± 53.93	372.8 ± 57.03
1,000 mg/kg BW	149.60 ± 8.87	148.60 ± 12.32	7.14 ± 0.42	7.94 ± 0.51	8.02 ± 0.83	8.11 ± 1.13	384.60 ± 58.82	362.00 ± 80.19
5,000 mg/kg BW	149 ± 12.5	145.66 ± 9.41	7.71 ± 0.50	7.98 ± 0.76	7.82 ± 0.73	8.19 ± 0.53	384.00 ± 49.52	398.60 ± 54.04

No statistically significant differences were found. CMC-Na, carboxyl methyl cellulose sodium; HG, hemoglobin concentration; RBC, red blood cell count; WBC, white blood cell count; PLT, platelet count; F, female; M, male.

**Table 4:** The effects of FA on serum biochemical parameters of rats in the 60-day subchronic toxicity assay.

Parameters	Gender	0.5% CMC-Na	200 mg/kg BW	1,000 mg/kg BW	5,000 mg/kg BW
30th day					
ALT (U/L)	F	44.89 ± 3.74	43.89 ± 3.34	48.84 ± 4.45	47.20 ± 6.88
	M	46.02 ± 5.78	46.03 ± 4.97	48.804.09	48.78 ± 6.74
AST (U/L)	F	178.34 ± 14.14	177.59 ± 3.49	169.26 ± 8.88	195.74 ± 21.70
	M	200.46 ± 27.01	186.33 ± 7.11	194.14 ± 41.96	21799 ± 30.3
BUN (mmol/L)	F	8.50 ± 0.83	8.47 ± 0.48	8.34 ± 0.36	8.49 ± 0.58
	M	8.48 ± 0.53	8.76 ± 0.33	8.62 ± 0.60	8.54 ± 0.35
Cr (umoL/L)	F	53.27 ± 7.11	53.60 ± 1.83	53.24 ± 2.26	54.92 ± 5.82
	M	44.84 ± 5.06	46.29 ± 3.92	45.86 ± 5.05	43.43 ± 3.19
Glu (mmol/L)	F	6.75 ± 0.77	6.80 ± 0.47	6.71 ± 0.38	6.16 ± 0.64
	M	6.6 ± 0.42	6.80 ± 0.43	6.70 ± 0.56	6.87 ± 0.77
Alb (mmol/L)	F	44.54 ± 1.58	44.16 ± 0.67	43.52 ± 2.77	43.91 ± 3.33
	M	42 ± 2.69	43.22 ± 2.05	37.82 ± 4.91	40.36 ± 1.93
TP (G/L)	F	81.03 ± 1.14	79.74 ± 1.75	79.48 ± 2.06	77.40 ± 3.92
	M	76.36 ± 4.38	73.1 ± 3.8	76.23 ± 6.22	74.48 ± 3.41
TCH (mmol/L)	F	3.18 ± 0.24	3.12 ± 0.15	3.19 ± 0.12	2.00 ± 0.65
	M	2.4 ± 0.39	2.53 ± 0.33	2.36 ± 0.61	2.45 ± 0.23

60th day					
ALT (U/L)	F	46.70 ± 5.12	53.60 ± 5.04	50.86 ± 3.35	50.20 ± 2.39
	M	45.40 ± 8.09	48.09 ± 5.49	50.00 ± 5.00	48.20 ± 3.03
AST (U/L)	F	205.48 ± 24.80	195.50 ± 32.31	177.59 ± 3.49	175.02 ± 14.14
	M	203.59 ± 17.76	186.48 ± 28.52	177.50 ± 10.64	185.42 ± 27.17
BUN (mmol/L)	F	8.22 ± 0.78	8.58 ± 0.32	8.47 ± 0.48	8.70 ± 0.66
	M	8.62 ± 0.72	8.96 ± 0.53	8.98 ± 0.29	8.90 ± 0.33
Cr (umoL/L)	F	49.52 ± 5.07	50.54 ± 12.05	53.10 ± 1.83	44.84 ± 5.06
	M	46.76 ± 3.97	47.02 ± 3.90	46.32 ± 3.91	46.32 ± 3.91
Glu (mmol/L)	F	6.76 ± 0.31	7.15 ± 0.82	7.03 ± 0.47	7.10 ± 0.51
	M	7.04 ± 0.30	7.10 ± 0.98	6.80 ± 0.43	6.90 ± 0.46
Alb (mmol/L)	F	40.86 ± 5.07	37.82 ± 4.91	3.46 ± 0.67	38.78 ± 2.13
	M	43.21 ± 3.95	42.03 ± 4.47	40.98 ± 2.05	41.98 ± 2.05
TP (G/L)	F	75.24 ± 4.91	74.78 ± 7.42	76.54 ± 2.87	77.98 ± 2.76
	M	74.86 ± 7.74	72.87 ± 2.94	71.38 ± 2.10	73.78 ± 5.49
TCH (mmol/L)	F	2.65 ± 0.53	3.08 ± 0.26	3.19 ± 0.15	3.30 ± 0.39
	M	2.45 ± 0.23	2.76 ± 0.47	2.60 ± 0.38	2.72 ± 0.31

No statistically significant differences were found. CMC-Na, carboxyl methyl cellulose-sodium; ALT, alanine aminotransferase; AST, aspartate aminotransferase; BUN, blood urea nitrogen; Cr, creatinine; Glu, glucose; Alb, total albumin; TP, total protein; TCH, total cholesterol. F, female; M, male.

**Table 5:** Results of the bacterial reverse mutation test of FA.

Drugs	Concentration (*μ*g per plate)	TA97		TA98		TA100		TA102	
		+S9	−S9	+S9	−S9	+S9	−S9	−S9	+S9
FA	1.6	156.0± 9.6	145.7 ± 9.3	42.7 ± 4.7	38.7 ± 4.5	165.7 ± 9.1	155.7 ± 6.1	284.3 ± 10.6	294.0 ± 8.2
	8	135.0 ± 8.5	148.0 ± 10.5	41.7 ± 2.5	41.3 ± 3.5	167.3 ± 8.0	157.0 ± 7.5	276.0 ± 6.6	284.3 ± 10.0
	40	153.3 ± 6.5	135.3 ± 8.0	37.3 ± 3.5	42.7 ± 4.2	174.7 ± 10.7	175.7 ± 12.6	277.3 ± 9.1	275.7 ± 9.9
	200	154.3 ± 8.5	137.0 ± 9.0	39.3 ± 4.0	37.3 ± 4.5	165.7 ± 8.6	172.7 ± 10.6	275.3 ± 8.5	306.0 ± 11.1
	1,000	163.0 ± 10.5	143.3 ± 10.1	34.0 ± 3.1	36.7 ± 3.1	159.3 ± 11.7	154.7 ± 6.5	283.3 ± 10.7	304.0 ± 10.0

H_2_O	—	143.7 ± 8.7	134.0 ± 7.0	39.0 ± 4.0	38.3 ± 4.2	174.3 ± 8.0	155.0 ± 12.1	267.0 ± 7.5	286.7 ± 11.1
DMSO	—	155.3 ± 7.1	128.0 ± 9.2	35.3 ± 3.8	38.0 ± 4.6	155.0 ± 7.5	166.7 ± 13.1	264.0 ± 7.0	257.0 ± 14.5
2-Aminoﬂuoren	10	898.3 ± 68.1^*∗*^	—	580.0 ± 28.2^*∗*^	—	920.0 ± 64.4^*∗*^	—	1329.0 ± 41.6^*∗*^	—
Sodium azid	1.5	—	—	—	—	—	1034.7 ± 57.9#	—	—
p-dimethyl amino benzene diazo sodium sulfonate	50	—	553.7 ± 31.1^*∗*^	—	559.3 ± 15.4^*∗*^	—	—	—	1380.7 ± 28.0^*∗*^

FA, fulvic acid; —, no data; #*p* < 0.01, compared to the corresponding control group; ^*∗*^*p* < 0.01, compared to the DMSO control group.

**Table 6:** The effects of FA on mouse bone marrow micronucleus and PCE/RBC ratio.

Gender		Animals	PCE/RBC radios	PCE micronucleus (%)
Female	Control	5	0.95 ± 0.07	1.20 ± 1.85
	1250	5	0.99 ± 0.08	1.39 ± 2.29
	2500	5	0.99 ± 0.14	1.32 ± 1.80
	5000	5	0.97 ± 0.15	1.30 ± 2.12
	CP	5	0.52 ± 0.04^*∗*^	21.34 ± 6.41^*∗*^

Male	Control	5	1.01 ± 0.08	1.34 ± 1.83
	1,250	5	0.98 ± 0.05	1. 14 ± 1.74
	2,500	5	0.98 ± 0.08	1.16 ± 1.78
	5,000	5	0.97 ± 0.06	0.95 ± 1.81
	CP	5	0.54 ± 0.06^*∗*^	23.46 ± 8.59^*∗*^

FA, fulvic acid; CP, cyclophosphamide; RBC, red blood cells; PCE, polychromatic erythrocytes; ^*∗*^*p* < 0.01, compared to the control group.

**Table 7:** Sperm abnormality test for FA in mice.

Parameters	Group (mg/kg)				
	Control	1,250	2,500	5,000	CP
Number of mice	5	5	5	5	5
Number of sperm observed	5 × 1000	5 × 1000	5 × 1000	5 × 1000	5 × 1000
Number of sperm abnormality	102	88	98	96	432
Abnormal ratio (%)	2.08 ± 0.53	1.79 ± 0.89	2.00 ± 0.78	1.96 ± 0.87	9.47 ± 1.96^*∗*^

Abnormal sperms counted ratio (%)					
No hook	11.46 ± 0.57	12.24 ± 0.68	12.50 ± 0.60	12.75 ± 0.59	7.18 ± 1.32
Banana shape	13.54 ± 0.79	10.20 ± 0.51	12.50 ± 0.83	13.73 ± 0.66	5.32 ± 0.99
Amorphous	42.71 ± 0.39	42.86 ± 1.55	44.32 ± 1.05	47.06 ± 0.49	56.25 ± 1.86
Large round head	11.46 ± 0.21	12.24 ± 0.60	10.23 ± 0.51	6.86 ± 0.49	9.72 ± 1.86
Kinks tail	5.21 ± 0.73	7.14 ± 0.59	4.55 ± 0.41	5.88 ± 0.47	3.47 ± 0.64
Two heads	2.08 ± 1.00	3.06 ± 0.37	5.68 ± 0.44	1.96 ± 0.31	2.78 ± 0.75
Two tails	13.54 ± 0.80	12.24 ± 0.75	10.23 ± 0.51	11.76 ± 0.60	15.28 ± 2.32

Cyclophosphamide (CP) is as a positive control; ^*∗*^*p* < 0.01, compared to the control group.

## Data Availability

All data used are included within the article.

## Supplementary Materials

Supplementary Materials Figure S1 The changes in average body weight gain (a), food efficiency (b), and daily food intake (c) in the 60-day repeated-dose oral toxicity studies of FA.
