# Supplementary material for: A Comprehensive Toxicological Assessment of Fulvic Acid
Source: Evid Based Complement Alternat Med. 2020 Dec 16;2020:8899244. doi: 10.1155/2020/8899244 (PMC7758121; doi:10.1155/2020/8899244)
Supplement: Supplementary Materials — Figure S1 The changes in average body weight gain (a), food efficiency (b), and daily food intake (c) in the 60-day repeated-dose oral toxicity studies of FA. [file 8899244.f1.docx]

B

A

Male

Female

C

Male

Female

Male

Female

Suppl. Figure 1 The changes of average body weight gain (A), food efficiency (B) and daily food intake (C) in the 60-day repeated-dose oral toxicity studies of FA.
